# Incorporation of Aramids into Polybenzimidazoles to Achieve Ultra-High Thermoresistance and Toughening Effects

**DOI:** 10.3390/molecules29051058

**Published:** 2024-02-28

**Authors:** Xianzhu Zhong, Aniruddha Nag, Kenji Takada, Akinori Nakajima, Tatsuo Kaneko

**Affiliations:** 1 Key Laboratory of Synthetic and Biological Colloids, Ministry of Education, School of Chemical and Material Engineering, Jiangnan University, 1800 Lihu Avenue, Wuxi 214122, China; zhongxz@jaist.ac.jp; 2 Graduate School of Advanced Science and Technology, Japan Advanced Institute of Science and Technology (JAIST), 1-1 Asahidai, Nomi 923-1292, Ishikawa, Japan; nag.aniruddha@ms.naist.jp (A.N.); takada@jaist.ac.jp (K.T.); blaziken257.larva@docomo.ne.jp (A.N.)

**Keywords:** configuration, polybenzimidazoles, thermal degradation, mechanical properties

## Abstract

Polybenzimidazoles (PBIs) are recognized for their remarkable thermal stability due to their unique molecular structure, which is characterized by aromaticity and rigidity. Despite their remarkable thermal attributes, their tensile properties limit their application. To improve the mechanical performance of PBIs, we made a vital modification to their molecular backbone to improve their structural flexibility. Non-*π*-conjugated components were introduced into PBIs by grafting meta-polyamide (MA) and para-polyamide (PA) onto PBI backbones to form the copolymers PBI-*co*-MA and PBI-*co*-PA. The results indicated that the cooperation between MA and PA significantly enhanced mechanical strain and overall toughness. Furthermore, the appropriate incorporation of aromatic polyamide components (20 mol% for MA and 15% for PA) improved thermal degradation temperatures by more than 30 °C. By investigating the copolymerization of PBIs with MA and PA, we unraveled the intricate relationships between composition, molecular structure, and material performance. These findings advance copolymer design strategies and deepen the understanding of polymer materials, offering tailored solutions that address thermal and mechanical demands across applications.

## 1. Introduction

Polybenzimidazoles (PBIs) are a distinctive class of polymers renowned for their exceptional thermal stability [1,2], a trait arising from their unique molecular architecture characterized by high aromaticity and inherent rigidity [3,4,5,6]. This structural arrangement has firmly established PBIs as formidable contenders for applications requiring resilience to extreme temperatures and challenging environmental conditions [7]. In particular, we established a process to prepare 2,5-PBI films, and the resulting derivatives are regarded as the most thermoresistant polymers on record [8]. This thermoresistance can be attributed to PBIs’ highly conjugated structure, comprising densely arranged aromatic benzene rings and hetero rings [9]. Despite their thermoresistance, this entirely aromatic molecular structure, at the same time, often exhibits a high rigidity, imparting an inherent inflexibility that results in a tensile elongation lower than 5%, below desired benchmarks, significantly restricting the mechanical toughness of PBIs [10,11,12]. As a result, the application of PBIs as high-performance polymer materials is greatly restrained [13].

The intersection of mechanical and thermal performance is of paramount importance across a diverse array of applications [14,15]. In the context of high-speed engines, a coating material is indispensable for its components, which operate under conditions characterized by extremely high temperatures and rapid rotary speeds [16,17]. Consequently, the requisite coating material necessitates an extraordinary combination of ultra-high thermal stability and toughness to withstand the extreme environmental conditions inherent in such applications. However, the majority of commercially available materials prove inadequate in meeting these stringent requirements. This shortfall is primarily attributed to the inherent limitations of many organic materials, which often exhibit relatively low thermal degradation temperatures. Even among engineering plastics, a notable category of materials, the majority are ill-equipped to endure temperatures exceeding 300 °C [18,19,20]. As such, the quest for materials possessing the requisite thermal stability and mechanical resilience for demanding applications persists as a formidable challenge within the realm of materials science and engineering.

PBIs exhibit initial thermal degradation approximately at 400 °C, surpassing the 600 °C threshold for 10% weight loss [21]. This characteristic aligns seamlessly with the demand for materials demonstrating exceptional thermal stability in environments of extreme temperatures. This underscores the need for the improvement of their mechanical elongations to achieve balanced and versatile performances in thermal stability and mechanical toughness. Consequently, the discordance between exceptional thermal resilience and relatively modest mechanical elongation has prompted researchers to explore innovative strategies for enhancing the overall mechanical attributes of these polymers [22,23,24]. For instance, diverse modifications have been implemented, encompassing the grafting of external functional groups with the objective of augmenting their free volume and mitigating rigidity [25,26]; the incorporation of components such as montmorillonite has been explored to create composite materials aimed at enhancing mechanical tensile elongations [27]. While these approaches have generally contributed to increased elongation, the improvements have been moderate and not exceptional. Importantly, these interventions have introduced a significant trade-off, notably compromising the thermal stability of the material [28,29]. Therefore, the optimization of tensile elongations without the concomitant sacrifice of thermal stability remains a formidable challenge.

In this study, the strategic integration of specific components, para-polyamide (PA) and meta-polyamide (MA), within the PBI matrix emerged as a promising pathway to potentially enhance mechanical elongation. The interplay between these supplementary components and the pre-existing PBI framework yields outcomes that intricately intertwine mechanical and thermal attributes, thereby contributing nuanced dimensions to the behavior of the polymer. Delving into the copolymerization of PBI with these specific components not only reveals the relationships between composition, molecular structure, and material performance, but also provides profound insights into the molecular interactions that underpin these properties. Consequently, the incorporation of PA and MA introduces a non-aromatic component into the PBI backbone that causes a decrease in tensile strength; however, owing to the improvement in molecular flexibility, the tensile elongation and overall toughness are significantly enhanced compared to the original PBI. In particular, the thermal stability is not compromised as the mechanical elongation is optimized. The findings of this study advance the design strategies of copolymers and enrich the broader understanding of polymer materials, leading to the creation of tailored solutions that effectively address the multifarious demands of thermal and mechanical stability across a wide spectrum of applications.

## 2. Results and Discussions

### 2.1. Polymer Preparations

Making a simple adjustment, such as attaching side functional groups to the backbone or creating a PBI composite, leads to a subsequent compromise in thermal stability. Therefore, we explored the incorporation of other functional polymers into PBIs to produce copolymers with enhanced mechanical tensile elongations. Polyamides were chosen due to their distinctive structure comprising both aliphatic aramid and aromatic benzene rings, rendering them compatible with PBIs without significantly altering their physical properties. We selected the meta- and para-structured precursor monomers MABA and PABA to examine the impact of bent and straight molecular structures on the resulting copolymers. 

The polymerization process was carried out in a highly acidic atmosphere using polyphosphoric acid (PPA) to expedite the reaction. To prevent the oxidation of amino groups with the rising temperature, precursor monomers were prepared in their hydrochloride form. Throughout the reaction, the viscosity of the reaction mixture exhibited a progressive increase, attributable to the concurrent elevation in the molecular weight of the copolymers. The resultant copolymers demonstrated a notable lack of solubility in nearly all conventional solvents, with the exception of strong acids. This insolubility can be attributed to the heightened reactivity of the amino groups present in precursor monomer3,4-diaminobenzoic acid (DABA), the collaboration of these amino groups during polymerization yields copolymers characterized by a substantial molecular weight. Additionally, the aromatic benzene rings and heterocycles impart a pronounced rigidity to the polymer structure. As a result, the copolymer exhibits significant resistance to dissolution in standard solvents.

The viscosity of the resultant polymers demonstrates a declining trend with an increase in the proportion of PA or MA. This phenomenon arises from the inherent flexibility of the aliphatic aramid in PA or MA, which increases the overall flexibility in comparison to the imidazole moiety present in PBIs. Moreover, the distinct reactivity of the amino groups in DABA, 4-aminobenzoic acid (PABA), and 3-aminobenzoic acid (MABA) may introduce hindrances to the cyclization process of imidazole, thereby leading to a reduction in molecular weight.

**Figure 1:**
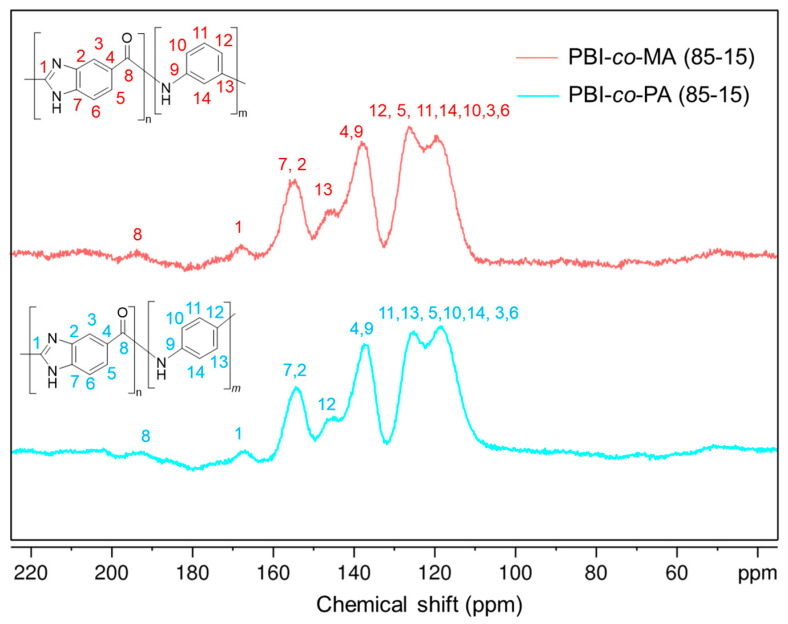
^13^C solid-state NMR (CP-TOSS) spectra of PBI-*co*-PA (above) and PBI-*co*-PA (down); the composition of both copolymers is 85-15 (mol%).

The structural confirmation of both copolymers was conducted through solid-state NMR, given their insolubility in regular solvents. Figure 1 depicts the spectra of copolymers with a composition of 85–15 (mol%) as a representative example. Notably, no significant differences were observed in the spectra, owing to the similar structure of MA and PA, with the only distinction being the meta and para positions of the bonded groups. The carbon atom assignments are specifically delineated in the spectra.

For both PBI-*co*-MA and PBI-*co*-PA, identical peaks were observed in the range of 195 to 130 ppm. The weak signal around 195 ppm was assigned to carbon 8, the only carbon not involved in the aromatic cycle; the signal at approximately 172 ppm was attributed to carbon 1, the imidazole carbon; the peak at around 154 ppm corresponded to carbon 7 and 2, the carbons shared by imidazole and benzene rings; the faint signal at 145 ppm was assigned to the carbon in the aramid benzene ring connected to imidazole; and the signal at approximately 138 ppm was attributed to the benzene rings involved in aramid.

However, in the range from 130 to 110 ppm, slight differences in signal strength were observed. PBI-*co*-MA exhibited two broad signals with peaks located at 126 and 120 ppm, respectively. The former peak was notably stronger, attributed to the meta-bonded structure of the aramid benzene ring, resulting in a distinct chemical shift difference among carbons. Specifically, carbons at positions 11, 12, and 14 exhibited a closer chemical shift higher than that of carbon 10, making the signals at 126 ppm significantly stronger. In contrast, the para structure of PBI-*co*-PA resulted in a relatively balanced chemical shift, with carbons at positions 11, 13, 10, and 14 displaying identical chemical shifts, respectively.

### 2.2. Mechanical Properties

**Figure 2:**
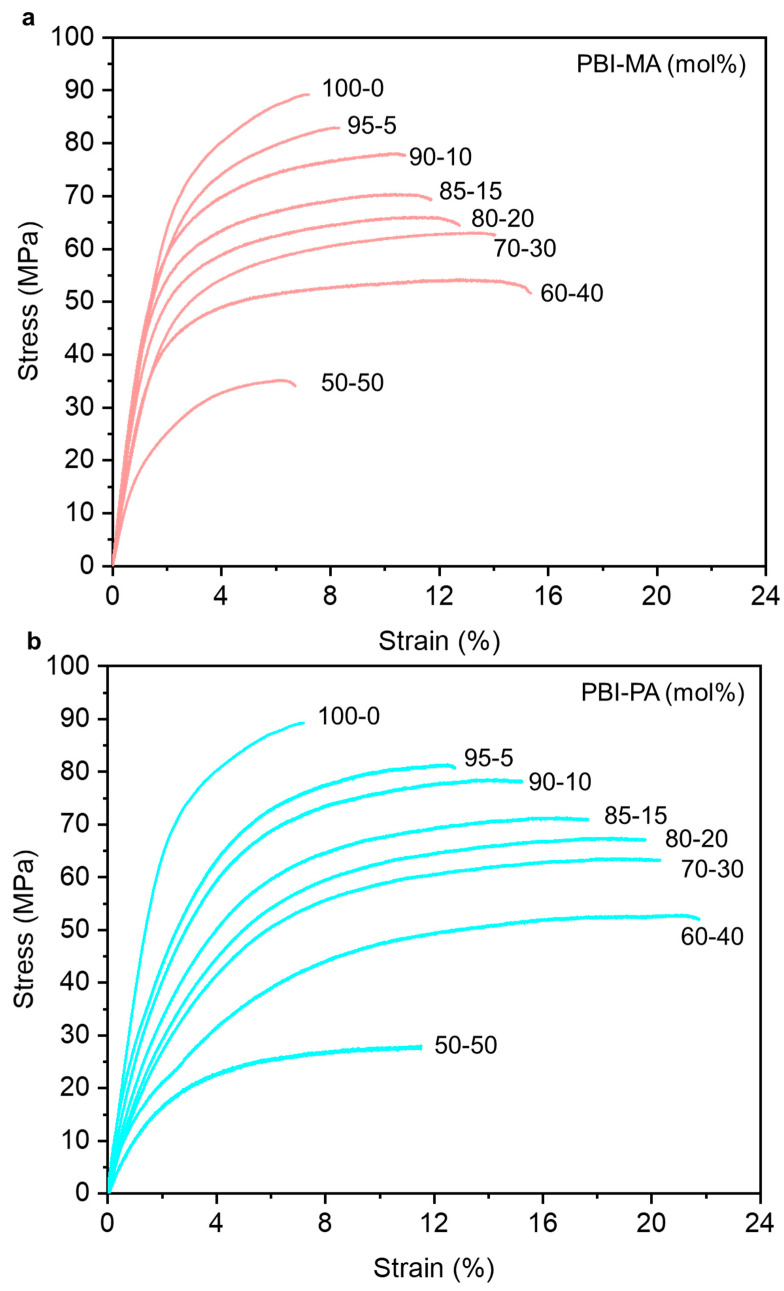
Stress–strain curves of PBI-*co*-MA (**a**) and PBI-*co*-PA (**b**) polymers.

**Figure 3:**
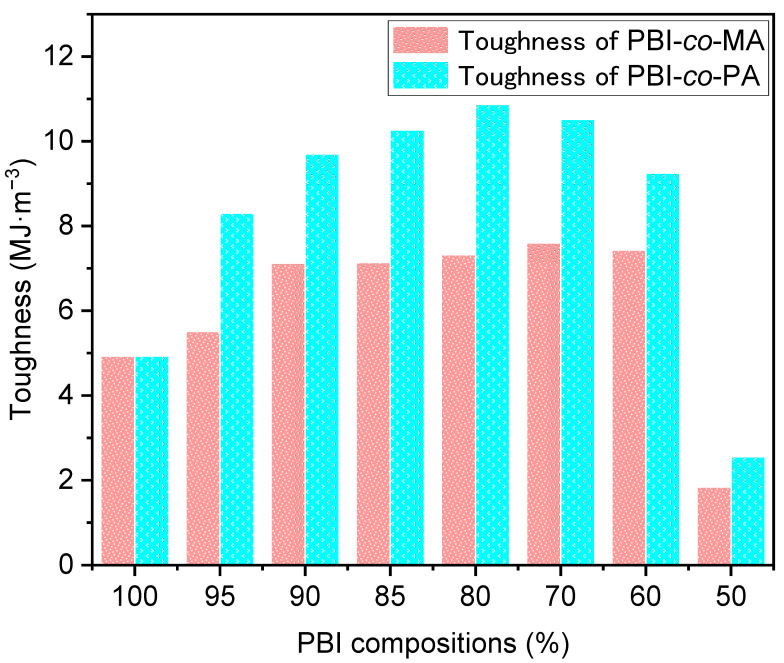
Toughness of PBI-*co*-MA and PBI-*co*-PA.

The copolymers PBI-*co*-PA and PBI-*co*-MA were synthesized using previously reported procedures, and their homogeneous films with smooth surfaces were processed for mechanical characterization. The mechanical characteristics of the copolymer films were assessed through stress–strain analysis using rectangular specimens, as depicted in Figure 2. The ultimate tensile strength, Young’s modulus, strain at break, and strain energy density (toughness) were evaluated; the specific values are listed in Table 1. Remarkably, the incorporation of MA and PA into the PBI backbone significantly affected the mechanical attributes of the materials. In contrast to the PBI homopolymer, the copolymers exhibited enhanced mechanical properties under certain conditions. In terms of the ultimate tensile strength, *s*, both copolymers exhibited a consistent decline from 89 MPa to approximately 52 MPa with molar compositions of MA or PA from 0% to 50%. Within the equivalent compositions, PBI-*co*-MA generally displayed a slightly superior strength compared to PBI-*co*-PA, as shown in Figure S1a. The Young’s modulus, *E*, also showed a decreasing trend with an increasing molar composition of MA or PA. Uniquely, the PBI-*co*-MA films were harder than the PBI-*co*-PA films. Conversely, PBI-*co*-PA displayed an increase in elongation at break, *e*, from 7.1% to 21.9% in a PBI composition range from 100% to 60%. However, in higher compositions, the *e* value decreased with an increase in composition. As a result, *e* exhibited a peak at a PA content of 40%. Similarly, *e* values of PBI-*co*-MA showed a peak at an MA content of 40%; however, the increasing degree was less than that in *e* of PBI-*co*-PA, as illustrated in Figure S1b. In accordance with the high value of elongation at break, the toughness, represented as the strain energy density, doubled as the MA or PA composition increased (Figure 3). The highest toughness values observed for PBI-*co*-MA and PBI-*co*-PA are 7.62 and 10.87 MJ·m^−3^, respectively. The addition of MA and PA effectively reduced the rigidity of the copolymers. However, when the composition reached 50%, the copolymers tended to become more brittle. This discrepancy can be ascribed to the distinctive structural configurations inherent in the two polymer variants. The non-aromatic aramid, by its nature, poses challenges in the fabrication of a ductile film. As the non-aromatic component starts to dominate the copolymer’s structural characteristics, the resultant texture transitions into a brittle state.

The non-aromatic structure of the aramid, in contrast to the rigid aromatic structure of the PBI, imparts greater flexibility to the copolymer backbone, contributing to the enhanced elongation observed with the incorporation of MA or PA. Theoretical considerations suggest that MA has the capacity to yield more substantial improvements in tensile elongations compared to polyamide (PA). This propensity arises from the unique bent configuration of MA, which introduces a higher degree of spatial freedom and increased free volume within its molecular structure. The bent geometry of MA facilitates enhanced flexibility and adaptability at the molecular level, potentially leading to superior tensile elongation performance. In contrast, the linear molecular structure of PA inherently offers fewer spatial degrees of freedom, limiting its ability to exhibit comparable improvements in tensile elongations. Nevertheless, the experimental results contradict the initial intuition, revealing that PA imparts consistently higher tensile elongations to the copolymer compared to MA. This unexpected outcome prompts an exploration of the underlying mechanisms, necessitating a comparative analysis of the three-dimensional molecular structures. 

**Figure 4:**
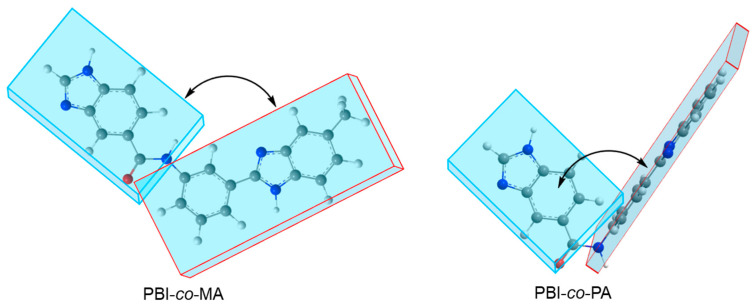
Single molecular configurations of PBI-*co*-MA (**left**) and PBI-*co*-PA (**right**).

As illustrated in Figure 4, the MM2 parameter in Chem 3D was used to analyze the structure of PBI-*co*-MA and PBI-*co*-PA; the inherent angle of the meta bond is apparent in the molecular structure. Despite this, the bent configuration resulting from the presence of a meta-bonded benzene ring is counteracted by the bent structure of the amino group. In contrast, PA initially possesses a linear structure without a discernible angle. 

However, upon assembly with imidazole, a notable dihedral angle emerges in the position of the aramid. This transformation results in a highly bent molecular configuration, showcasing a greater curvature than the plane angle formed by maleic anhydride (MA) and imidazole. The elevated curvature contributes to increased free volume and spatial degree of freedom, implying that PBI-*co*-PA films exhibit greater flexibility and pliability. Remarkably, this observation aligns with the experimental results, providing valuable insights into the structural basis for the mechanical properties observed in the copolymer system.

### 2.3. Thermal Decomposition

**Figure 5:**
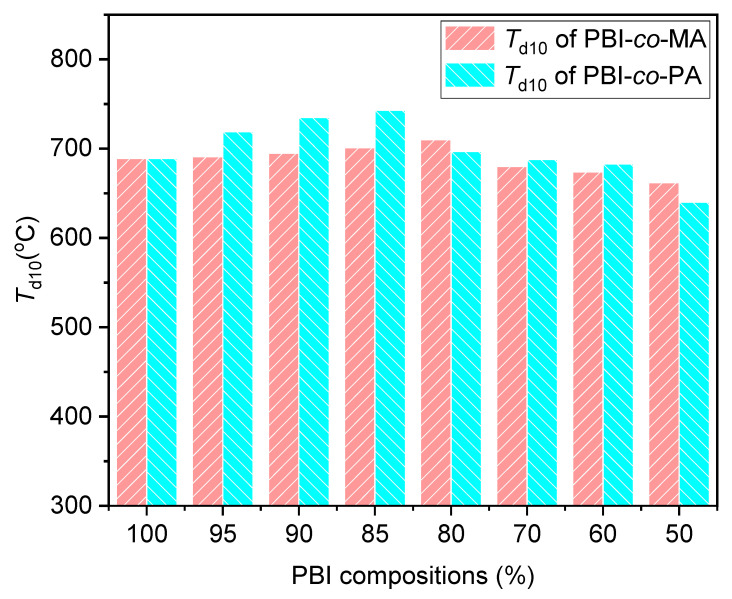
*T*_d10_ values of PBI-*co*-PA and PBI-*co*-MA copolymers.

Both PBI-*co*-PA and PBI-*co*-MA exhibited impressive thermoresistance, as evidenced by their respective 10% mass loss temperatures (*T*_d10_), surpassing most of the known organic materials with mass loss temperatures below 600 °C. Their TGA curves are shown in Figures S2 and S3. The distinctive behavior of these copolymers, with respect to their thermal performances under varying compositions of MA and PA, reveals intriguing insights into the delicate interplay among compositions and material properties (Figure 5). Conventionally, as the content of lower thermoresistance units within higher thermoresistance copolymers increases, a decline in the overall thermoresistance can be expected. However, the effects of small amounts of PA or MA within the PBI framework on the thermoresistance of the resulting materials are more complex than expected. While the *T*_d10_ of the PBI homopolymer was 689 °C, that of PBI-*co*-PA with a PA content of 15% showed an enhanced value of 743 °C, suggesting a synergistic effect between the original PBI composition and the introduced PA. Nevertheless, a subsequent decline became notable as the PA content exceeded 15%, decreasing *T*_d10_ values. An analogous phenomenon was observed from the PBI-*co*-MA copolymer; however, with an MA composition of 80%, the *T*_d10_ attains a summit at 710 °C, which is lower than that of PBI-*co*-PA. At an MA composition above 80%, the *T*_d10_ decreased with an increase in MA content. Similar to the pattern observed with PA, a subsequent gentle decline ensues as the MA content is held at 50%, resulting in a *T*_d10_ of 640 °C.

This intricate phenomenon can be rationalized by delving into backbone rigidity and molecular interactions. The incorporation of PA or MA into the copolymer matrix intrinsically decreases backbone rigidity, as evidenced by the mechanical elongation ability, which decreases thermal stability owing to enhanced mobility. However, such unwanted effects on thermal resistance are negligible when the incorporation degree is small. As previously reported [7], the incorporation of a small number of aramid units enhanced the interaction enthalpy, mainly the hydrogen bonding of imidazoles and/or amides. Notably, the resilient resonance effect inherent in aromatic polymer chains undergoes perturbation upon the incorporation of aramids. This perturbation, arising from the dispersive nature of the aramid moieties, introduces a degree of electron delocalization that disrupts the resonant electron cloud, consequently modulating the charge distribution along the chain. Specifically, electrons associated with hydrogen or nitrogen atoms are released from the confines of the resonance system, culminating in an environment conducive to interchain hydrogen bond formation [30]. Consequently, the integration of polyamide significantly augmented the interchain hydrogen bonds, thereby constricting the molecular chains and amplifying the interaction energy among polymers. These influences ultimately culminated in a copolymer that exhibited greater durability when exposed to high temperatures. This resilience is evident in the higher *T*_d10_ of the copolymers compared to the homopolymer.

Nevertheless, with a further increase in the proportion of PA or MA, a critical point is reached. Beyond a specific threshold (specifically, 85% for PA and 20% for MA), the flexibility and mobility inherent in aramids start to govern the properties of the molecular chains. As aramids feature an aliphatic structure with lower thermal stability than an aromatic structure, they begin to compromise the integrity of the molecular chain, making it susceptible to decomposition at elevated temperatures. This detrimental impact of aramids becomes more pronounced than the positive influence of strengthening hydrogen bonds. Consequently, an excessive content of aramids leads to a decline in the thermal stability of the copolymers. 

Consequently, the incorporation of a small amount of aramid units, such as PA and MA, into the PBI backbone enhanced the thermostability; however, the effects were limited because of the intrinsically low thermostability of PA or MA. The intricate orchestration of these factors collectively shapes the thermal response of copolymers, shedding light on the delicate equilibrium between structural modifications and inherent material attributes. 

## 3. Materials and Methods

### 3.1. Materials

3,4-diaminobenzoic acid (DABA), 3-aminobenzoic acid (MABA), 4-aminobenzoic acid (PABA), and sodium hydroxide (NaOH) were procured from TCI (Tokyo Chemical Industry, Tokyo, Japan). Poly(phosphoric acid) (PPA) with 85% purity was sourced from Sigma-Aldrich. Methanesulfonic acid (MSA) and trifluoroacetic acid (TFA) were provided by Wako Pure Chemical Industries, Ltd. (Osaka, Japan) The pH test paper used in this research was supplied by Macherey-Nagel GmbH & Co. KG, Düren, Germany. All the solvents and reagents employed in this study were utilized as received without any additional processing or purification. 

### 3.2. Hydrochlorization of Monomers

DABA (1.5 g, 10 mmol) was dissolved in methanol (50 mL) and immersed in an ice bath. Approximately 1 mL of 12 N hydrochloric acid was added dropwise to the solution until the powder completely dissolved. The mixture was then stirred at 25 °C for 24 h. Afterward, the white-colored salt of 3,4-diaminobenzoic acid dihydrochloride (DABA·2HCl) was obtained through solvent evaporation (yield: 2.11 g, 95%). MABA·HCl and PABA·HCl were obtained using the same chloritization method. 

### 3.3. Syntheses

**Scheme 1:**
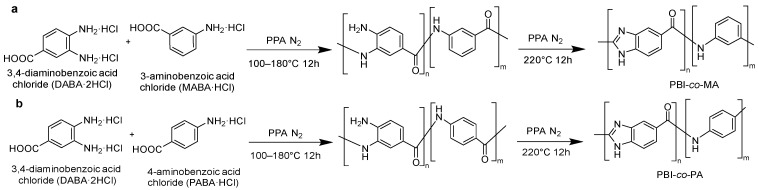
Synthetic pathway of (**a**) PBI-*co*-PA and (**b**) PBI-*co*-MA.

An example of the synthesis of all polymers is shown using PBI-*co*-PA in an 85-15 mol% ratio. Initially, 40 mg PPA was placed in a three-necked round-bottomed flask equipped with a magnetic stirrer. The flask was heated at 100 °C for 1 h under a nitrogen gas atmosphere to remove any residual moisture. Subsequently, DABA·2HCl (940.8 mg, 4.2 mmol) and PABA·HCl (3.1 mg, 0.18 mmol) were added to the flask. The contents were stirred continuously for 1 h until all the moisture was eliminated and the monomeric solids were completely dissolved in the reaction system. The mixture was then successively heated at 160, 180, and 200 °C for 4 h each, followed by a final heating step at 220 °C for 12 h. During this process, the color of the solution changed from red to dark brown. After completion, the resulting solution was dispersed in water and stirred for 12 h to remove PPA. Subsequently, a brown solid was obtained via filtration. The solid was then dried under a vacuum, ground into a powder, and suspended in deionized water. Afterward, 1 M NaOH was slowly added, and the solution was stirred until a pH of approximately 7.0 was reached, which was maintained for 1 h. The solid product was collected by filtration and dried under a vacuum, resulting in a brown powder (621.2 mg, 5.3 mmol) as the final product with a yield of 89%. The same synthetic process was repeated for each polymer with different molar ratios and for all PBI-*co*-MA series of polymers. The synthetic pathway is shown in Scheme 1. 

### 3.4. Film Fabrication

**Figure 6:**
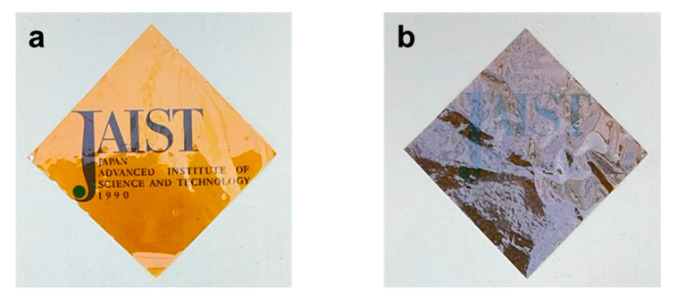
Images of PBI copolymer films. (**a**) PBI-*co*-PA molar composition 85-15; (**b**) PBI-*co*-MA molar composition 80-20.

A solution of the PBI-*co*-PA copolymers (100 mg) in 3 mL of trifluoroacetic acid (TFA) and one droplet of methanesulfonic acid (MSA) was cast onto a silicon substrate. The film was dried at 25 °C, after which it was gently detached from the substrate and immersed in deionized water for 24 h to eliminate lingering solvents. The resulting film was then subjected to further drying at 80 °C for 12 h, ultimately yielding a pliable film (Figure 6a). A PBI-*co*-MA film (Figure 6b) was cast using an analogous procedure for PBI-*co*-PA. When the MA or PA exceeded 50%, the film became too brittle to characterize. 

### 3.5. Measurements

Solid-state 13C NMR CP/TOSS spectra of the terpolymers were acquired using a Bruker Advance III spectrometer operating at 500 MHz. The terpolymer samples were loaded into a 7 mm diameter zirconia rotor with a Kel-F cap and spun at 8 kHz. The contact time and period between successive accumulations were set to 2 and 5 s, respectively. In total, 25,000 scans were obtained.

The viscosity of the terpolymer was assessed using a SIBATA 026300-3 viscometer, with concentrated sulfuric acid (H_2_SO_4_) employed as the solvent. Thermogravimetric analysis (TGA) of the terpolymers was performed using a HITACHI STA7200. The samples (5 mg) were placed in a platinum crucible and heated to 1000 °C under a nitrogen atmosphere at a heating rate of 10 °C/min. The thermal decomposition temperatures were determined by measuring the 10% weight loss temperature (*T*_d10_) of the samples.

The stress–strain curves of the terpolymers were obtained through tensile-mode mechanical tests using an Instron-3365 mechanical tester at room temperature. The samples were shaped into a rectangular film with a 40 mm length, 7 mm width, and 15 μm thickness. The elongation speed was set to 0.4 mm/min.

## 4. Conclusions

During our ongoing research on the synthesis of high-performance polymers, aromatic amino acids were used to prepare polybenzazoles and aramids. In this study, we synthesized PBI-*co*-PA and PBI-*co*-MA copolymers across a range of compositions. Some of these synthetic approaches yielded films with heightened mechanical toughness compared to the PBI homopolymer. Notably, the distinctive bent configuration inherent in the MA structure did not impart a commensurate increase in elasticity to the copolymer because of the compensation arising from the aramid structure. Conversely, despite the linear alignment of the PA molecule attributed to para-bonding, it retained the angular orientation of the aramid. This intricate configuration led to a relatively modest dihedral angle between the PBI and PA units, introducing enhanced chain tortuosity, and consequently contributing to elevated polymer elongation. Significantly, both the PBI-*co*-PA and PBI-*co*-MA copolymers showed commendable thermal stability, a feature that proved to be intricately entwined with the introduction of MA and PA components. Intriguingly, the judicious incorporation of these constituents increases the thermoresistance. This fortification can be attributed to the perturbation caused by the introduced components, which disrupts the resonance effect inherent in the PBI backbone. Moreover, this disruption amplified the interaction enthalpy among the molecular chains. These counterintuitive findings underscore the profound insights these investigations offer into molecular structure and configurations, thereby contributing to a broader understanding of polymer behavior.

## Figures and Tables

**Table 1:** Specific values of properties in various characterizations.

P ^a^ (%-%)	U ^b^ (MJ/m^3^)	*E* ^b^ (GPa)	*σ* ^b^ (Mpa)	*γ* ^b^ (%)	*η* ^c^ (dL/g)	*T*_d10_ ^d^ (°C)
PBI-MA 100-0	4.93	3.15	89.1 ± 2.35	7.1 ± 0.10	3.1	689
95-5	5.51	2.95	82.3 ± 4.05	8.2 ± 0.15	3.1	691
90-10	7.12	3.03	77.9 ± 2.31	10.8 ± 0.16	3.0	695
85-15	7.14	2.87	71.2 ± 2.06	11.8 ± 0.10	2.6	701
80-20	7.32	2.58	67.0 ± 3.34	12.7 ± 0.13	1.9	710
70-30	7.60	2.16	69.3 ± 3.60	14.0 ± 0.21	1.5	680
60-40	7.43	2.20	52.5 ± 2.91	15.1 ± 0.10	1.2	674
50-50	1.84	1.35	27.6 ± 3.08	5.5 ± 0.17	0.8	662
PBI-PA 100-0	4.93	3.15	89.1 ± 2.35	7.1 ± 0.10	3.1	689
95-5	8.30	2.13	82.6 ± 3.96	12.6 ± 0.22	3.0	719
90-10	9.70	1.96	77.6 ± 3.60	15.2 ± 0.15	2.8	735
85-15	10.27	1.52	70.1 ± 2.47	17.7 ± 0.15	3.3	743
80-20	10.87	1.35	65.0 ± 4.10	19.8 ± 0.18	2.5	697
70-30	10.52	1.28	62.7 ± 2.90	20.4 ± 0.10	2.1	688
60-40	9.25	1.10	52.8 ± 3.43	21.9 ± 0.15	1.5	683
50-50	2.55	0.90	34.8 ± 3.12	11.5 ± 0.21	1.1	640

^a^ Copolymers with varying PBI and MA/PA compositions. ^b^ Mechanical properties U, *E*, *σ*, and *γ*, measured by stress–strain tensile test, refer to toughness, Young’s modulus, tensile strength at break, and strain at beak, respectively. ^c^ Inherent viscosity of the copolymer. ^d^ Thermal property indices measured by TGA in a nitrogen atmosphere. Ten percent weight loss thermal decomposition temperature (*T*_d10_).

## Data Availability

Data are contained within the article and Supplementary Materials.

## Supplementary Materials

The following supporting information can be downloaded at: https://www.mdpi.com/article/10.3390/molecules29051058/s1, Figure S1: Mechanical indexes of PBI-*co*-MA and PBI-*co*-PA. (a) Tensile strength; (b) elongation at break; Figure S2: TGA curves of PBI-*co*-MA in various compositions. The curves show that the thermal degradation temperatures increase as the MA ratio increases to 20%, followed by a decreasing trend as MA ratios continue increasing; Figure S3: TGA curves of PBI-*co*-PA in various compositions. Similar to the curves of PBI-*co*-MA, the thermal degradation temperatures increase as the PA ratio increases to 15%, followed by a decreasing trend as the PA ratios continue increasing.
