# Supplementary material for: Incorporation of Aramids into Polybenzimidazoles to Achieve Ultra-High Thermoresistance and Toughening Effects
Source: Molecules. 2024 Feb 28;29(5):1058. doi: 10.3390/molecules29051058 (PMC10934712; doi:10.3390/molecules29051058)
Supplement: Supplementary file 1 [file molecules-29-01058-s001.zip › molecules-2843498-supplementary.pdf]

Incorporation of Aramids into Polybenzimidazoles to Achieve Ultra-High Thermoresistance and Toughening Effects

Xianzhu Zhong [1,2], Aniruddha Nag [1], Kenji Takada [1], Akinori Nakajima [1] and Tatsuo Kaneko [1,2,*]

[1] Key Laboratory of Synthetic and Biological Colloids, Ministry of Education, School of Chemical and Material Engineering, Jiangnan University, 1800 Lihu Avenue, Wuxi 214122, China; zhongxz@jaist.ac.jp (X.Z.); nag.aniruddha@ms.naist.jp (A.N.); takada@jaist.ac.jp (K.T.); blaziken257.larva@docomo.ne.jp (A.N.)

[2] Graduate School of Advanced Science and Technology, Japan Advanced Institute of Science and Technology (JAIST), 1-1 Asahidai, Nomi 923-1292, Ishikawa, Japan

* Correspondence: tkaneko@jiangnan.edu.cn

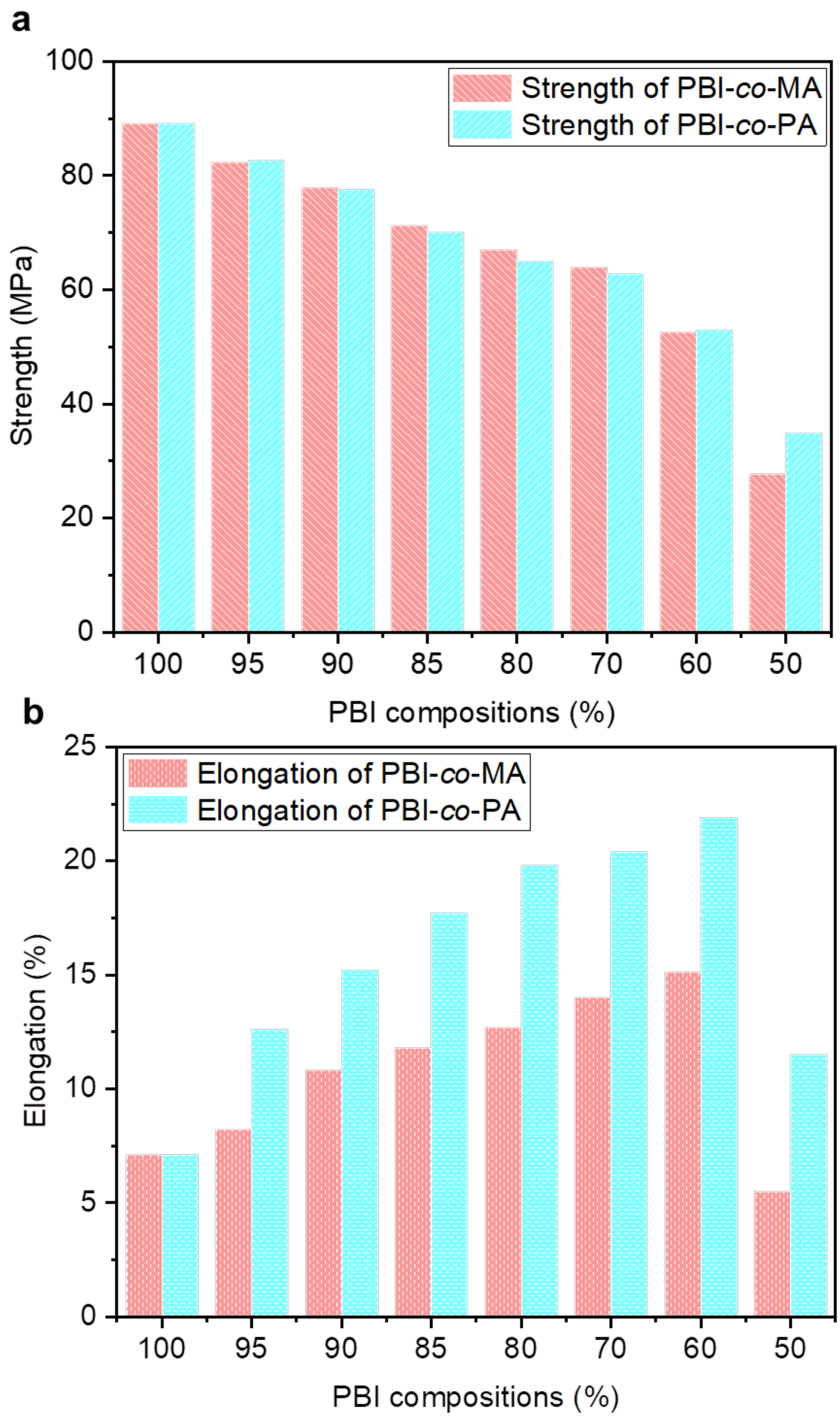

Figure S1. Mechanical indexes of PBI-*co*-MA and PBI-*co*-PA. a: tensile strength; b: elongation at break.

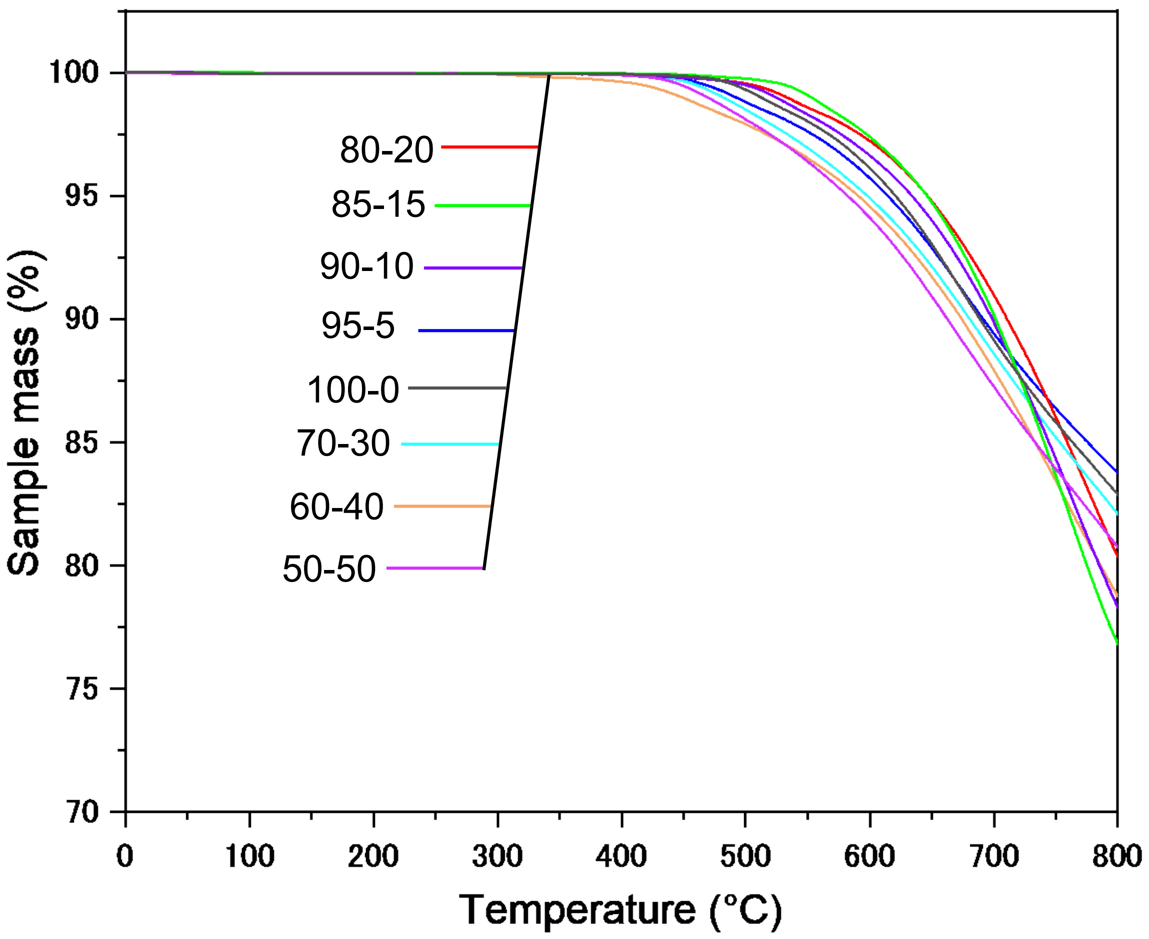

Figure S2. TGA curves of PBI-*co*-MA in various compositions. The curves shows that the thermal degradation temperatures increase as the MA ratio increases to 20%, followed by a decreasing trend as continuing increasing the MA ratios.

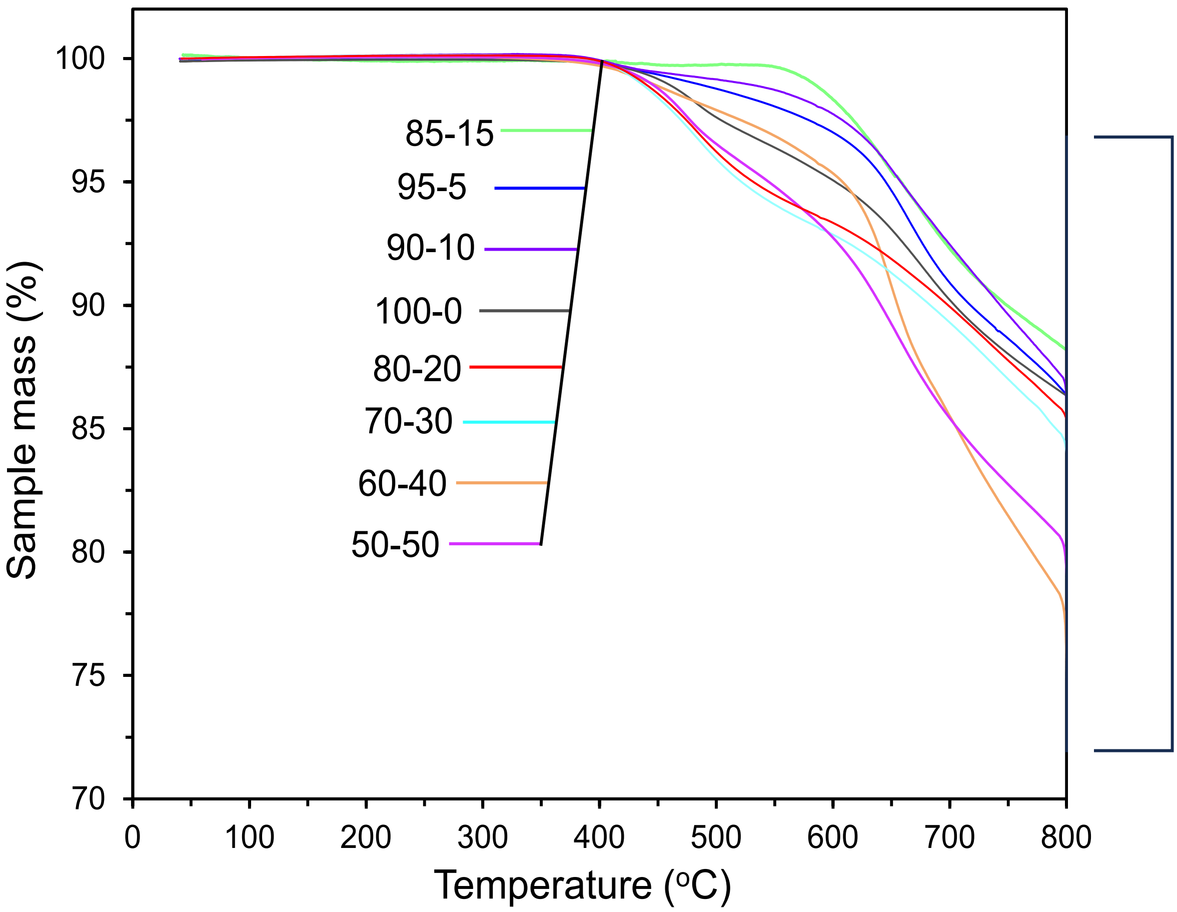

Figure S3. TGA curves of PBI-*co*-PA in various compositions. Similar as the curves of PBI-*co*-MA, the thermal degradation temperatures increase as the PA ratio increases to 15%, and followed by a decreasing trend as continuing increasing the PA ratios.
